# Molecular Surface Chemistry Drives Anomalous Clustering of Ultrasmall Silica Nanoparticles

**DOI:** 10.1021/acs.jpclett.6c01796

**Published:** 2026-07-09

**Authors:** Ruchi Patel, Gernot Rother, Noshir Pesika, Greg A. Kimmel, Yan Levin, Gregory K. Schenter, Christopher J. Mundy, Jaehun Chun, Bhuvnesh Bharti

**Affiliations:** † Cain Department of Chemical Engineering, 5779 Louisiana State University, Baton Rouge, Louisiana 70803, United States; ‡ Neutron Scattering Division, 6146 Oak Ridge National Laboratory, Oak Ridge, Tennessee 37831, United States; § Department of Chemical and Biomolecular Engineering, 5783 Tulane University, New Orleans, Louisiana 70115, United States; ∥ 6865 Physical and Computational Sciences Directorate, Pacific Northwest National Laboratory, Richland, Washington 99354, United States; ⊥ Instituto de Física, 133642 Universidade Federal do Rio Grande do Sul, Caixa Postal 15051, CEP 91501-970 Porto Alegre, RS, Brazil

## Abstract

Silica nanoparticles are expected to remain stable at alkaline pH because deprotonated silanol groups lead to a strong electrostatic interparticle repulsion. Here, we show that this mean-field expectation fails for ultrasmall silica nanoparticles. Electron microscopy, dynamic light scattering, and small-angle X-ray scattering (SAXS) show that 9 nm silica nanoparticles form finite equilibrium clusters at pH 8.7, whereas larger particles remain dispersed under identical conditions. Analysis of SAXS profiles demonstrate that interactions between smaller nanoparticles cannot be described by typical screened electrostatics alone and require an additional short-range attractive contribution. Surface-sensitive measurements show that decreasing particle size increases hydroxylated silanol groups, alters interfacial charge density, and enhances the contribution of higher-p*K*_a_ proton-active sites. The cluster size reaches a maximum when pH approaches the higher-p*K*_a_, identifying surface protonation state as a key descriptor. These results show that molecular surface chemistry drives pH-dependent attractions in ultrasmall silica nanoparticles beyond mean-field electrostatics.

## Graphical abstract

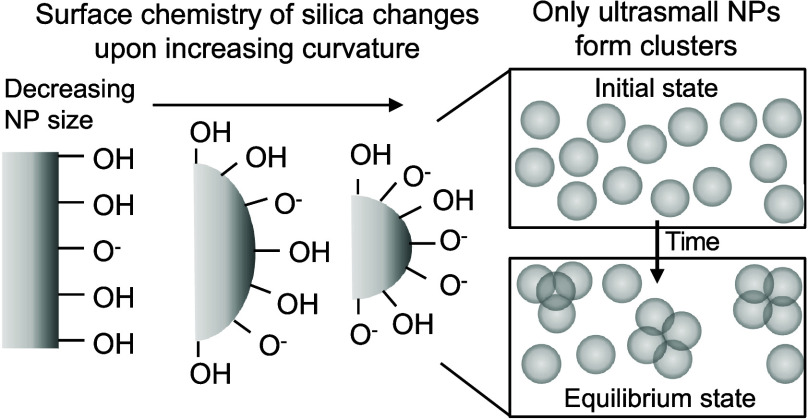

Understanding interparticle interactions is central to predicting the stability, and assembly of nanoscale materials in aqueous environments.^1,2^ Nanoparticles (NPs) become increasingly difficult to synthesize, disperse, and stabilize as their size approaches the ultrasmall sub-10 nm regime. This challenge is often attributed to their high surface-to-volume ratio, which amplifies interfacial contributions to the free energy of the dispersion, based on purely thermodynamic rationale.^3^ As particle dimensions decrease, interfacial forces may become increasingly sensitive to molecular-scale features of the surface, including heterogeneous chemistry, discrete ionizable sites, and local hydration.^1,4,5^ These features introduce interactions that are not fully captured by classical mean-field descriptions.^1,3,6,7^ Existing theoretical frameworks have incorporated molecular detail primarily in the solution phase through ion specificity, hydration, and solvent structure near solid–liquid interfaces,^8,9^ while treating the surface largely as a continuum boundary with averaged charge or potential. Whether this approximation remains sufficient for ultrasmall NPs, or whether the molecular nature of surface chemistry introduces unprecedented interactions beyond classical mean-field electrostatics, remains unresolved.

Classical electrostatic models describe NP interactions in aqueous environments through repulsion arising from osmotic pressure and electric Maxwell stress associated with overlapping double layers.^10^ This approach implicitly treats the surface charge on a NP as a quasistatic and uniformly distributed quantity at a given interparticle separation.^1^ However, surface charge is regulated by reversible ion adsorption and acid–base equilibria of interfacial functional groups, controlled by pH, ionic strength, and local chemistry.^11^ Although charge regulation is often adopted as a more realistic boundary condition, it is typically implemented using time-averaged, spatially uniform surface charge densities governed by bulk ionic composition and surface equilibria.^10,12^ This averaged description may be insufficient for ultrasmall NPs due to a relatively small number of ionizable groups involved in charging, especially near the effective p*K*_a_ of surface groups where protonation states are nearly degenerate. Under these conditions, local proton activity, hydrogen-bonding asymmetry, and surface-site heterogeneity may generate short-range attractions that are beyond the assumptions of conventional theories.

Here, we investigate whether ultrasmall silica NPs exhibit pH-dependent attractions that are not captured by classical mean-field electrostatics. Silica provides a well-controlled platform because its size can be varied systematically and its surface contains heterogeneous silanol functional groups that undergo acid–base regulation near their apparent p*K*_a_ values.^13–17^ We observe that ultrasmall silica NPs spontaneously form finite-sized equilibrium clusters under conditions where mean-field electrostatics predict repulsive interactions. This clustering is shown to be absent in larger silica NPs, which remain dispersed under identical solution conditions as predicted by classical theories. Small angle X-ray scattering (SAXS) structure-factor analysis shows that the interactions between the smaller NPs require an additional short-range attractive contribution, as well as screened electrostatic repulsion and van der Waals attraction. This new attraction depends on particle size and pH, with clustering maximized when the solution pH approaches the higher-p*K*_a_ silanol population. These results identify the protonation state of surface silanol groups as a key descriptor for the clustering and establish that ultrasmall silica NPs can access a pH-dependent attractive regime not captured by conventional mean-field descriptions, revealing anomalous collective responses in nanoparticle dispersions that originate from molecular-scale surface chemistry. The origin of this attraction remains unresolved, but its dependence on size, silanol chemistry, and acid–base equilibria points to molecular surface chemistry as the source of the departure from classical predictions.

## Clustering of Ultrasmall Silica NPs

We studied silica nanospheres with diameters of 9, 15, and 27 nm at pH 8.7, which is well above the known point of zero charge (PZC) of silica (PZC ∼ 3). We used commercially available silica NPs (Ludox W.R. Grace & Co.-Conn.) and determined their size distribution by transmission electron microscopy (TEM) (Figure S1). Before experiments, aqueous dispersions of silica NPs were dialyzed in deionized HPLC-grade water at pH ∼ 10 for 1 week to minimize the ionic strength of the continuous phase. After dialysis, dispersions containing 12 wt % (volume fraction ∼ 0.06) silica NPs were sonicated, adjusted to pH 8.7, and allowed to equilibrate for 90 days (see Materials and Methods in SI). The state of NPs after 90-day equilibration was examined using cryogenic-TEM (cryo-TEM), which shows that the smallest NPs (9 nm) exist as two populations, singlets and clusters (Figure 1A). In contrast, 27 nm silica NPs remain fully dispersed as singlets, even in the mixed dispersion where 9 nm NPs show clusters (Figure 1B–C).

**Figure 1:**
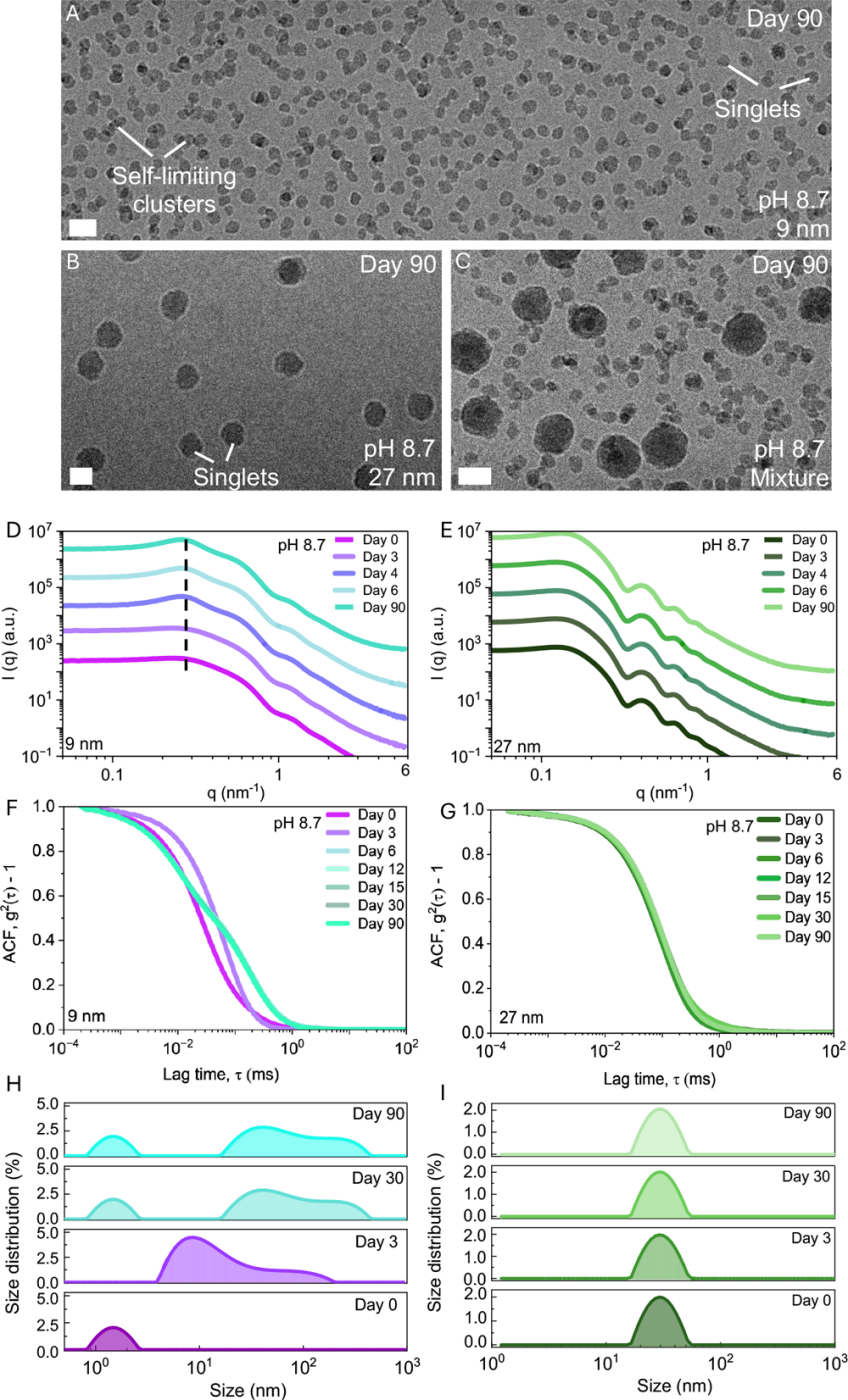
**Size-dependent formation of equilibrium clusters in silica NPs.** (A-C) Cryo-TEM images of silica NPs equilibrated at pH 8.7 for 90 days. The 9 nm NPs (A) show coexistence of isolated singlets and finite clusters, whereas larger 27 nm NPs (B) remain dispersed as singlets under identical conditions. In the mixture of NPs (C), clustering is observed exclusively for the 9 nm NPs. Scale bars in A−C represent 20 nm. (D-E) SAXS intensity profiles for 9 nm (D) and 27 nm (E) silica NPs measured after 0, 3, 4, 6, and 90 days of equilibration at pH 8.7. The curves are shifted upward by a factor of 10 for better visualization. For 9 nm silica, equilibration leads to the emergence of a pronounced low-*q* peak (dashed line) while preserving the form factor oscillations, indicating changes in the assembled state without alteration of particle geometry. In contrast, the SAXS profile of 27 nm NPs equilibrated at pH 8.7 remains unchanged. (F-G) Normalized ACFs from DLS during equilibration show that 9 nm NPs (F) develop two distinct relaxation times, indicating coexisting singlets and clusters, whereas 27 nm NPs (G) show minimal temporal evolution and retains single particle dynamics. (H–I) The size distributions obtained from ACFs show a transition from an initially unimodal to a bimodal distribution for 9 nm NPs, while 27 nm NPs remain unimodal throughout the equilibration period.

The temporal evolution of the assembled state was further examined using SAXS and dynamic light scattering (DLS). In the SAXS profile for 9 nm NPs, a structure factor peak emerges at *q* ∼ 0.3 nm^–1^ after 6 days of equilibration at pH 8.7, whereas the primary form factor oscillation at *q* ∼ 1 nm^–1^ remains unchanged (Figure 1D-E and Figure S2). This evolution indicates clustering without measurable changes in primary particle size or geometry.^18^ No changes in either form or structure factor were observed for the 27 nm NPs. At Day 0, all tested NP sizes showed DLS-based normalized intensity autocorrelation functions (ACFs) characteristic of well-dispersed individual NPs (Figure 1F-G and Figure S3), with unimodal intensity-weighted size distributions consistent with singlets (Figure 1H–I and Note S1). Upon equilibration at pH 8.7, the 9 nm dispersion developed two distinct relaxation times after Day 6, corresponding to coexisting singlets and clusters. The bimodal distribution persisted through 90 days, indicating a near-equilibrium population of finite clusters. The DLS-derived intensity-weighted size distribution should be interpreted with caution because clusters dominate the scattering signal. Thus, the apparent disappearance of singlets for 9 nm silica at Day 3 likely reflects the limited ability of DLS to resolve singlets from early clusters that remain close in size (Figure 1H). This interpretation is supported by SAXS, which shows no significant structure-factor peak at Day 3 (Figure S2). In contrast, 27 nm NPs remained fully dispersed throughout 90 days of equilibration (Figure 1I and SI). The intermediate-size silica NPs (15 nm) show a gradual increase in size with equilibration time, but this increase is less significant than that observed for 9 nm NPs (Figure S4). Both measurements show that finite clustering is strongly size-dependent and most pronounced for 9 nm silica NPs. Importantly, the clusters formed by 9 nm NPs were reversible upon increasing pH, with singlets recovered after raising the dispersion to pH ∼ 10.3 (Figure S5). At this pH, silica NPs remained dispersed irrespective of size (Figure S6), showing that clustering requires both ultrasmall particle size and the appropriate pH window.

To quantify the size dependence, we extracted approximate interactions from SAXS structure factors (Figure 2A-D). SAXS profiles for all three sizes were analyzed with independently determined form factors kept fixed during analysis (Figure S7 and Notes S2–S3). We tested two simple interaction models: (1) a one-Yukawa potential representing only screened repulsion, and (2) a two-Yukawa potential with competing attraction and repulsion. The one-Yukawa structure factor described by the Hayter-Penfold mean spherical approximation for charged spheres, uses independently measured surface charge and Debye length (∼3 nm) with no free fit parameters (Table S1). This structure factor quantitatively reproduces the SAXS profile of 27 nm particles (Figure 2A). However, increasing deviations appear as NP size decreases, most pronounced for 9 nm particles (Figure 2B–C), indicating that the screened repulsion alone is insufficient for the smaller particles. We therefore use a two-Yukawa potential of the form^19–21^

1 $$\frac{U\left(x\right)}{kT}=\frac{K_{1}e^{-Z_{1}\left(x-1\right)}}{x}+\frac{K_{2}e^{-Z_{2}\left(x-1\right)}}{x}$$

where *x* = *r*/2*a* and *r* is the center-to-center interparticle separation and *a* is the radius of the NP, *k* is the Boltzmann constant and T is the temperature. The first term represents the experimentally constrained screened repulsion with *K*_1_ (>0) and *Z*_1_ fixed from measured charge and ionic strength. The second term captures an additional short-range attraction with *K*_2_ (<0) and *Z*_2_ (>*Z*_1_) as free parameters (Table S2). The two-Yukawa model provides significantly improved representation of the scattering profiles for the 15 and 9 nm particles (Figure 2B–C). The resulting potentials show purely repulsive interactions for 27 nm particles, whereas smaller NPs exhibit an additional short-range attraction (Figure 2D). This attraction promotes NP clustering, while the longer-ranged repulsive barrier limits cluster growth and prevents macroscopic phase separation, consistent with self-limiting equilibrium clusters arising from competing short-range attraction and long-range repulsion.^22–27^

**Figure 2:**
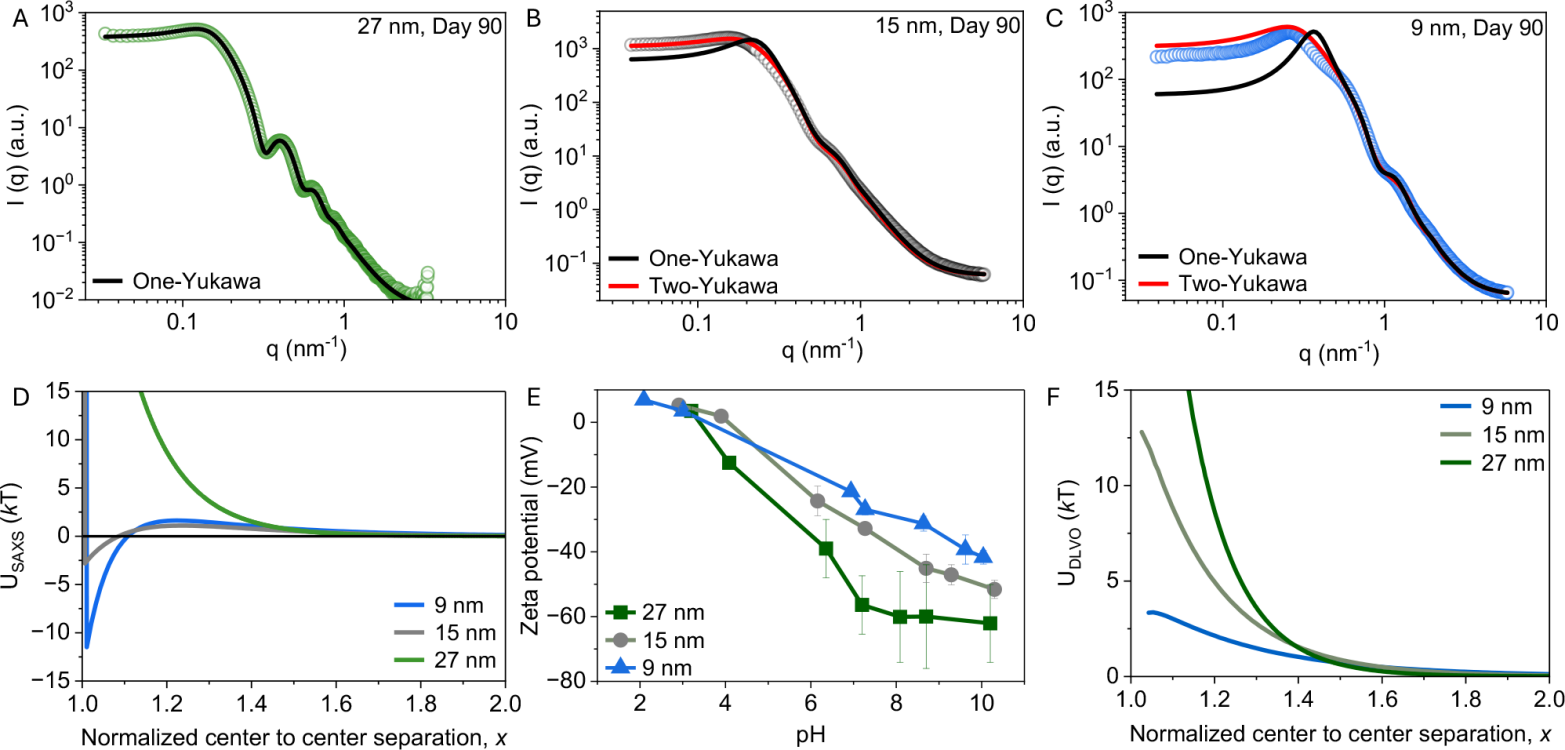
**Emergence of short-range attraction upon decreasing NP size.** (A-C) SAXS profiles of silica NPs (9, 15, and 27 nm diameter) equilibrated for 90 days at pH 8.7. Black curves show *a priori* predictions of scattering intensity (form and structure factor) using independently measured particle size, polydispersity, surface charge density, and ionic strength, modeled as polydisperse hard spheres interacting via a screened Yukawa (mean-field electrostatic) potential. Red curves show fits using a two-Yukawa potential structure factor with an additional short-range attractive component that is necessary to better represent the low-*q* structure factor peak observed for 15 and 9 nm NPs. (D) Effective pair potentials extracted from SAXS show purely repulsive interactions for largest NPs, whereas 9 nm particles exhibit a short-range attractive minimum followed by a longer-ranged repulsive barrier. (E) Change in zeta potential as a function of pH for 9 nm, 15 nm, 27 nm silica NPs. (F) Classical DLVO interaction energy as a function of center-to-center separation normalized by particle diameter for silica NPs of all sizes at pH 8.7, predicting purely repulsive interactions, which would lead to stable dispersions across all particle sizes, which is not the case as shown in (A-D).

The requirement for an additional attractive contribution is further supported by DLVO (Derjaguin–Landau–Verwey–Overbeek) theory, along with direct electrokinetic measurements. Zeta potential measurements show that all silica NPs remain negatively charged across the investigated pH range, with large negative potentials at pH 8.7 (Figure 2E). Thus, based on mean-field electrostatics alone, all three dispersions should be stabilized by double-layer repulsion. To test this expectation, we calculated the classical DLVO interaction energy by combining electrostatic double-layer repulsion with van der Waals attraction:^10,28^

2 $$U_{EDL}\left(x\right)=\frac{2\pi \varepsilon_{0}\varepsilon a\zeta^{2}e^{-2\kappa a\left(x-1\right)}}{x}$$

3 $$U_{vdW}\left(x\right)=-\frac{A}{6}\left[\frac{1}{2\left(x^{2}-1\right)}+\frac{1}{2x^{2}}+\ln\left(\frac{x^{2}-1}{x^{2}}\right)\right]$$

where κ is the inverse Debye length, and ζ is the measured zeta potential, and *A* is the Hamaker coefficient (Note S4). We use zeta potential in place of the surface potential because the effective electrostatic interaction is known to be governed by a potential at the hydrodynamic shear plane/outer Helmholtz region that more closely represents a “diffuse layer potential” than the potential at bare chemical surface^29–31^ (Note S5). The calculations were cut off at an interparticle separation of 0.3 nm because hydration, steric, ion-specific, and surface-roughness effects may become important at such molecular-scale separations,^32^ which are beyond the validity of continuum DLVO theory. This cutoff does not affect the conclusion because the interaction profiles remain repulsive across the evaluated range, indicating that the particles are not predicted to reach close contact. These calculations predict purely repulsive interaction profiles for all particle sizes at pH 8.7 (Figure 2F), inconsistent with the clustering observed for 9 nm silica NPs in cryo-TEM and the short-range attraction indicated by SAXS data analysis. Therefore, the experimentally observed clustering cannot be explained by conventional DLVO theory alone.

## Size-Dependent Surface Chemistry

Silica surfaces contain fully condensed siloxane sites (Q^4^) and multiple hydroxylated silanol groups, including geminal (Q^2^), isolated (Q^3a^) and vicinal (Q^3b^) silanols, each associated with distinct local bonding, hydrogen-bonding, and acid-base characteristics^33^ (Figure 3A). To quantify how these surface group populations vary with particle size, we used ^29^Si Cross-Polarization Magic-Angle Spinning Nuclear Magnetic Resonance (CP-MAS NMR, Figure 3B) and vibrational spectroscopy (FTIR, see SI).^34,35^ Analysis of the NMR and FTIR spectra shows that decreasing particle size increases Q^2^, Q^3a^, and Q^3b^ silanol populations, with a corresponding reduction in Q^4^ groups (Figure 3B,C and Note S6). These results show that smaller silica NPs present a more hydroxylated interface, enriching proton-active silanol groups at the expense of condensed siloxane sites.

**Figure 3:**
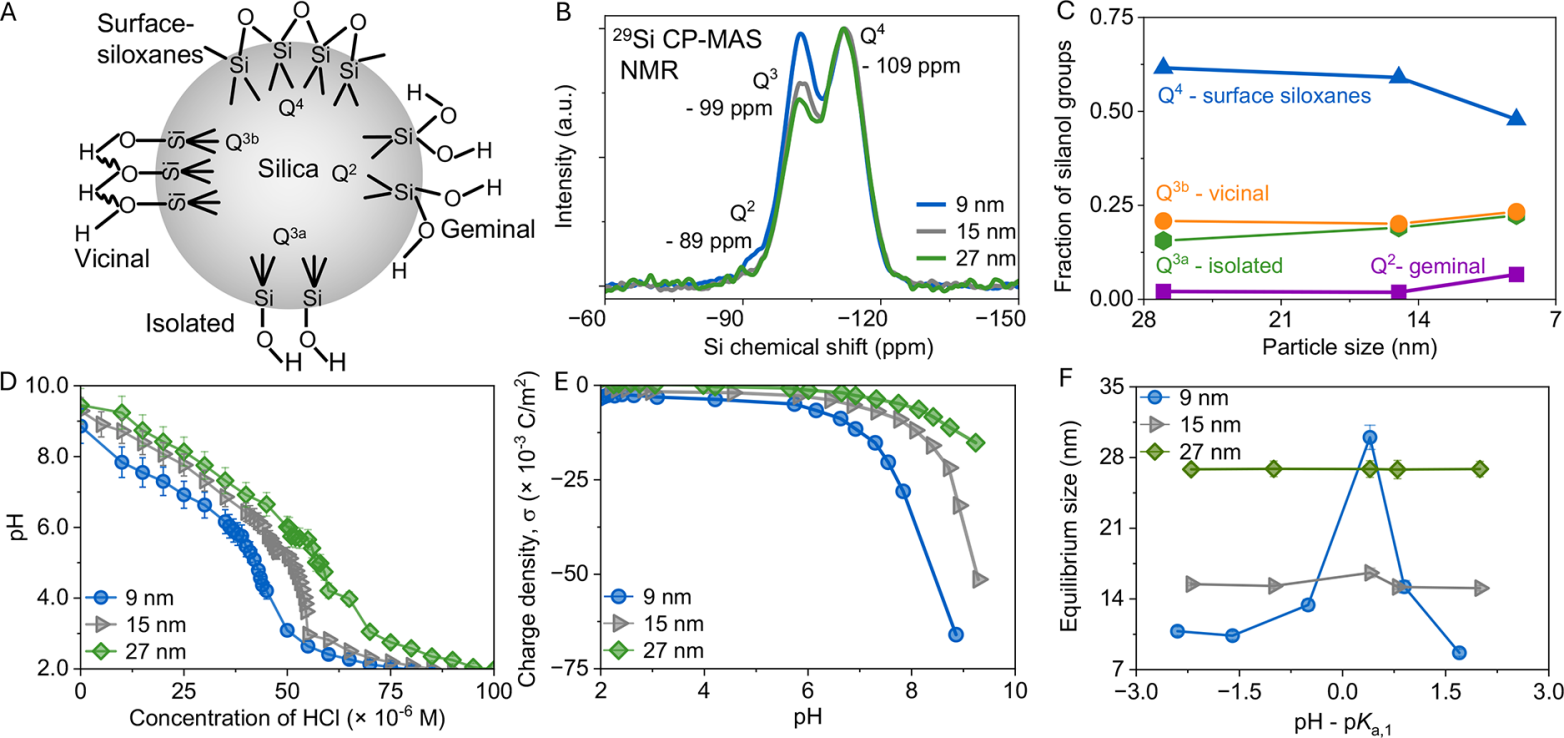
**pH dependent charge density and surface-chemistry of silica NPs**. (A) Schematic showing the diversity in the surface chemical groups on silica NP surface which include siloxane (Q^4^) sites and hydroxylated silanol species (Q^2^, Q^3a^ and Q^3b^) with distinct acid–base properties. (B) ^29^Si CP-MAS NMR spectra for 9, 15, and 27 nm particles, showing systematic size-dependent changes in Q^2^, Q^3^ (Q^3a^ and Q^3b^) and Q^4^ silicon groups. (C) Quantified fractions of silanol and siloxane species derived from NMR and vibrational spectroscopy, showing an increase in isolated and vicinal silanol groups and a corresponding decrease in siloxane content with decreasing NP size. (D) Potentiometric titration curves for silica NP dispersions. (E) Surface charge density calculated from the titration data for 9, 15, and 27 nm particles; smallest NPs show significantly higher surface charge density than the largest one. (F) Equilibrium cluster sizes as a function of pH - p*K*_a,1_, with a pronounced maximum near pH - p*K*_a,1_ = 0 (±1), identifying the regime where the short-range attractive interaction becomes influential, leading to observed clustering in the 9 nm silica, unlike larger NPs.

The increase of hydroxylated Q^2^ and Q^3^ functional groups is reflected directly in the proton uptake and surface charging behavior measured by potentiometric titration. All three NP dispersions show pH-dependent proton uptake/release characteristic of ionizable silanol groups, with titration curves consistent with a two-step acid–base behavior of silica (Figure 3D). We define p*K*_a,1_ = 8.3 as the apparent acidity of the high-p*K*_a_ silanol population, associated primarily with isolated Q^3a^ and external geminal Q^2^ silanols (Note S7), and p*K*_a,2_ ∼ 4.5 as the apparent acidity of the lower-p*K*_a_ population, associated primarily with vicinal Q^3b^ and internal geminal Q^2^ silanols.^36,37^ Converting the titration curves to surface charge density shows that the 9 nm NPs carry the highest interfacial charge density over the alkaline pH range, including near pH 8.7 where clustering is observed (Figure 3E; Note S8). Thus, the clustered 9 nm NPs are not weakly charged particles approaching charge neutralization. Instead, they possess substantial negative surface charge that should, in a conventional mean-field electrostatic description, enhance NP stabilization. Therefore, clustering cannot be explained by a simple loss of electrostatic repulsion, and the size-dependent molecular surface chemistry; i.e., distribution of discrete silanol groups must be included to understand the effective interactions between ultrasmall silica NPs.

The increased abundance of Q^3a^ and external Q^2^ silanol groups in smaller NPs increases the contribution of p*K*_a,1_ (higher-p*K*_a_) proton-active sites, shifting the effective acid–base response of the interface toward the alkaline pH range. As a result, the pH 8.7 condition at which 9 nm silica NPs cluster lies close to p*K*_a,1_ = 8.3, making the relevant control parameter not simply pH or surface charge density, but the proximity of the solution pH to the acid–base equilibrium of the dominant silanol population. This connection is captured by plotting the equilibrium cluster size against pH - p*K*_a,1_ where the 9 nm silica NPs exhibit a pronounced maximum near pH - p*K*_a,1_ = 0 (Figure 3F). Outside this regime, particles remain dispersed due to dominant electrostatic double-layer repulsion or form bulk aggregates when van der Waals attraction dominates (Figure S8). The fact that this dependence appears only for the smallest NPs indicates that, in the ultrasmall size regime, the number, identity, and spatial distribution of discrete ionizable surface groups become central to the effective interparticle interaction. These results provide direct experimental evidence that molecular nature of charge-regulating sites can produce measurable deviations from mean-field electrostatic predictions.

The SAXS-derived structure factors demonstrate the existence of a short-range attraction for the smallest nanoparticles that cannot be explained by the conventional DLVO theory. The multimodal experiments identify the following three characteristics of the attraction: (i) it depends on the acid–base state of the silica–water interface and is maximized near pH - p*K*_a,1_ = 0, with an implicit dependence of p*K*_a_ on the particle size, (ii) the effective range of the attraction is shorter than the far-field electrical double layer repulsion but longer than that of van der Waals attraction, resulting in an attraction with ∼2–3 nm separation for the smallest nanoparticles, and (iii) clustering diminishes as the particle size increases.

Considering these characteristics, one possible rationale for the unknown attraction is proton-mediated charge fluctuations proposed by Kirkwood and Shumaker (KS).^38^ According to the classic KS model to describe protein aggregation, at pH → p*K*_a_ of the surface functional groups, where the ionizable states that can take on values of −1, 0, or +1 (in electronic charge unit) become nearly degenerate, leading to surface charge fluctuations. These resulting charge fluctuations produce transient dipole moments (μ) with nonzero variance, i.e., ⟨Δμ^2^⟩ ≠ 0, which can drive an effective attraction between notionally neutral surfaces.^39^ Using the same analysis on charged nanoparticles with silanol populations fluctuating from 0 to −1 predicts an attractive contribution that increases near the silanol p*K*_a_ (Note S9) and is inversely proportional to square of particle size, qualitatively consistent with the experimental trends. However, unlike in the case of proteins, for silica nanoparticles the leading order mean-field (DLVO) repulsive term is dominant,^6^ making the clustering unlikely to be characterized by the classical description of KS.^38^

An additional hypothesis for a short-range attraction is due to “frustrated” hydrogen bonding.^40,41^ Silanol groups on a highly curved surface could reorient nearby water molecules, forming a locally frustrated hydrogen-bonding network that could plausibly induce a short-ranged attraction. These molecularly structured hydration layers have been implicated for both positive and negative attraction between large semi-infinite surfaces in pure water.^40^ Additional complexities occurring in highly curved nanoparticles include local variations in charge density, steric accessibility, and the relative orientations of neighboring surface groups that may differ substantially from those on planar or weakly curved surfaces. Therefore, explaining the observed clustering based purely on hydrogen bonding becomes confounded at the scales considered in this study, and perhaps solvation-mediated effects in conjunction with charge regulation are necessary to overcome the electrostatic repulsion. Future investigations into how the curvature of 9 nm silica nanoparticles influence the interfacial solvent and local electrostatic environment remain important directions to pursue. In summary, our experiments identify a weak short-range attraction between ultrasmall silica nanoparticles that is coupled to surface protonation states, while leaving its molecular origin and the kinetic lag time required to reach quasi-equilibrium cluster sizes as open questions.

We show that ultrasmall silica NPs form finite equilibrium clusters under conditions where conventional DLVO theory predicts stable, repulsive dispersions. SAXS structure-factor analysis reveals the existence of an additional short-range attraction for the smaller NPs, while the clustering cannot be explained by loss of electrostatic repulsion. The attraction is linked to silica molecular surface chemistry; decreasing NP size enriches hydroxylated silanol groups, increases interfacial charge density, and shifts the effective acid–base equilibria. The cluster size reaches a maximum near pH - p*K*_a,1_ = 0, identifying the protonation state of silanol groups as a key descriptor for clustering. These results demonstrate that molecular-scale surface chemistry and discrete charge-regulating sites drive a pH-dependent attractive regime in ultrasmall silica NPs beyond mean-field electrostatic predictions.

## Supplementary Material



## Notes

Experimental materials and methods; DLS, cryo-TEM, SAXS, zeta potential, NMR, FTIR, conductivity, titration, and ICP-OES analyses; estimation of nanoparticle size distributions, dispersion salinity, pair interaction potentials, DLVO interaction energies, surface charge density, silanol speciation, intrinsic silanol p*K*_a_, and silanol dissociation free energy; supplementary figures showing nanoparticle size distributions, pH-dependent conductivity and salinity, NMR/FTIR-based silanol speciation, p*K*_a_ estimation, reversibility of clustering, time-dependent DLS clustering behavior, SAXS structure factor parameters, and residual ion concentrations (PDF)
