# Supplementary material for: Molecular Surface Chemistry Drives Anomalous Clustering of Ultrasmall Silica Nanoparticles
Source: J Phys Chem Lett. 2026 Jul 9;17(29):8270–6. doi: 10.1021/acs.jpclett.6c01796 (PMC13403300; doi:10.1021/acs.jpclett.6c01796)
Supplement: Supplementary file 1 [file jz6c01796_si_001.pdf]

Supplementary Information for

Molecular Surface Chemistry Drives Anomalous Clustering of Ultrasmall Silica Nanoparticles

Ruchi Patel[1], Gernot Rother[2], Noshir Pesika[3], Greg A. Kimmel[4], Yan Levin[5], Gregory K. Schenter[4], Christopher J. Mundy[4], Jaehun Chun[4,*], and Bhuvnesh Bharti[1,*]

[1]*Cain Department of Chemical Engineering, Louisiana State University, Baton Rouge, LA 70803, USA*

[2]*Neutron Scattering Division, Oak Ridge National Laboratory, Oak Ridge, TN, 37831, USA*

[3]*Department of Chemical and Biomolecular Engineering, Tulane University, New Orleans, Louisiana 70115, USA*

[4]*Physical and Computational Sciences Directorate, Pacific Northwest National Laboratory, Richland, WA 99354, USA*

[5]*Instituto de Física, Universidade Federal do Rio Grande do Sul, Caixa Postal 15051, CEP 91501-970 Porto Alegre, RS, Brazil*

*Corresponding authors: jaehun.chun@pnnl.gov and bbharti@lsu.edu

1. Materials and Methods

1.1. Materials

Following are the details of the chemicals used in this study, including supplier and purity: Ludox SM, Ludox HS, and Ludox TMA silica nanoparticles (W.R. Grace & Co.-Conn.), Hydrochloric acid (HCl, W.R. Grace & Co.-Conn, 5 N); Sodium hydroxide pellets (NaOH, W.R. Grace & Co.-Conn, ≥95%) and HPLC-grade water (H_2O, Fisher Scientific).

1.2. Methods

i. Preparation of silica nanoparticle dispersions

Silica nanoparticle dispersions were prepared using commercially available Ludox SM, Ludox HS, and Ludox TMA particles with TEM diameters of approximately 9 nm, 15 nm, and 27 nm, respectively. The nanoparticles were purified prior to experiments by dialysis against HPLC-grade water for one week with twice-daily water exchange to remove residual ions and foreign molecules from the dispersions. After dialysis, the dispersions were filtered through 220 nm syringe filters and sonicated to eliminate larger aggregates. The concentration of all dispersions was adjusted to 12 ± 0.66 wt%, the pH was adjusted to ~ 10 to avoid aggregation of silica and the dispersions were stored at 8 °C.

ii. Cryogenic Transmission Electron Microscopy (Cryo-TEM)

Dialyzed Ludox SM and Ludox TMA silica nanoparticle dispersions were prepared in HPLC-grade water at ~12 wt. %, and sonicated after adjusting the pH to 8.7. The samples were allowed to equilibrate for 90 days and were vitrified prior to imaging to preserve the structure of the aqueous dispersion. Cryogenic transmission electron microscopy (cryo-TEM) was used to directly visualize the equilibrium structure of the dispersions. A 4 μL aliquot of each sample was applied to 200-mesh copper grids with a lacey carbon support film, blotted with filter paper for 2 seconds, and rapidly vitrified in liquid ethane. Images were acquired using a Tecnai G2 F30 TWIN transmission electron microscope operated at an accelerating voltage of 120 kV and a temperature of about −170 °C.

iii. Small-Angle X-ray Scattering (SAXS)

SAXS measurements were performed on aqueous dispersions of Ludox SM, Ludox HS, and Ludox TMA dialyzed in HPLC-grade water. The dispersions were adjusted to pH 8.7 and sonicated prior to loading into capillary cells (~2 mm diameter). The samples were allowed to equilibrate in the capillaries for durations appropriate to the kinetic study before measurement.

Majority of the measurements were carried out at the synchrotron SAXS beamline at the Center for Advanced Microstructures and Devices (CAMD, LSU), which employs a double-crystal monochromator with an energy range of approximately 5 - 11 keV. Data reduction and de-smearing were performed using SasView software and the fitting was performed using appropriate models.

iv. Dynamic Light Scattering (DLS)

For DLS measurements, the pH of the dialyzed and filtered silica stock dispersions was adjusted by adding 1 M HCl or 1 M NaOH using a pre-calibrated pH electrode (Hanna Instruments HI 4112). After pH adjustment, approximately 3 mL of the dispersion was transferred into sealed plastic cuvettes for measurements. Measurements were conducted using an Anton Paar Litesizer 500 (Anton Paar GmbH) equipped with a 40 mW He-Ne laser (λ = 658 nm) and a detector positioned at a scattering angle of 90°. Samples were equilibrated for 2 minutes at 22 °C prior to measurements, and for each sample, 10 measurements consisting of 30 runs were performed to obtain the intensity-weighted autocorrelation function, $g^2(\tau) - 1$, as a function of lag time τ.

v. Zeta potential measurements

Dispersions of silica nanoparticles with mean diameters of 9 nm,15 nm, and 27 nm were prepared in HPLC-grade water at the desired pH. The samples were equilibrated for 2 min at 22 °C before starting the measurements. Measurements of electrophoretic mobility were carried out with Anton Paar Litesizer 500, Anton Paar, GmbH instrument operating with a 40 mW He-Ne laser (658 nm) in a Univette cuvette measurement cell. Six measurements, each consisting of 30 runs, were performed for each sample. The zeta potential, ζ, was further obtained from the measured electrophoretic mobility, μ_e.

vi. ^{29}Si CP-MAS nuclear magnetic resonance (NMR) spectroscopy

Solid-state ^{29}Si nuclear magnetic resonance (NMR) spectroscopy was used to determine the distribution of Q-species on the silica nanoparticle surface. Aqueous dispersions of dialyzed silica nanoparticles were first dried at 80 °C to remove bulk water and obtain dry powders suitable for solid-state NMR measurements. The resulting powders were gently ground and packed into zirconia MAS rotors. ^{29}Si spectra were acquired using cross-polarization magic-angle spinning (CP-MAS) on a Bruker AV spectrometer using a 9.4 T wide bore magnet and a 4 mm MAS probe, with a spinning rate of 4 kHz, a 10 ms cross-polarization contact time, and a 5 s relaxation delay. The data were processed using exponential multiplication with 100 Hz line

broadening, and the chemical shift scale was referenced to DSS at 0.0 ppm. Under these conditions, the ^{29}Si spectra exhibit characteristic resonances corresponding to different Q-species environments of silica. Silicon atoms bonded to two siloxane bridges and two hydroxyl groups (geminal silanols, Q^2) appear at ~ −89 ppm, silicon atoms bonded to three siloxane bridges and one hydroxyl group (single silanols, Q^3) appear at ~ −99 ppm, and fully condensed siloxane environments (Q^4) appear at ~ −109 ppm[1]**.** The experimental spectra were analyzed by Gaussian peak deconvolution of the total spectral envelope. Three Gaussian components corresponding to Q^2, Q^3, and Q^4 environments were fitted to the experimental spectra after baseline correction. The integrated area of each component was used to quantify the relative population of the corresponding Q-species[1]. The fractional abundance of each species was calculated by normalizing the area of each fitted peak to the total integrated spectral intensity (for details see Supplementary Note S6).

vii. Fourier transform infrared (FTIR) spectroscopy

Fourier transform infrared (FTIR) spectroscopy was used to probe the local environment of specifically the Q^3 silanol groups on silica nanoparticles. Prior to measurement, aqueous dispersions of the dialyzed nanoparticles were dried at 80 °C to remove bulk water and obtain solid samples. FTIR spectra were recorded using the Nicolet iS-50 FTIR spectrophotometer (Thermo Fisher Scientific) with a spectral resolution of 4 cm^{-1}, using transmission mode. Silica surfaces exhibit several characteristic hydroxyl stretching modes depending on the hydrogen-bonding environment of the silanol groups. Isolated silanol groups produced a sharp absorption band near 3750 cm^{-1}**,** whereas hydrogen-bonded silanols**,** often referred to as vicinal silanols, appeared at lower wavenumbers near 3660 cm^{-1} due to the weakening of the O-H bond through intermolecular hydrogen bonding[2] (for details see Supplementary Note S6). The FTIR spectra of the silica nanoparticles were analyzed by examining the relative intensities of the isolated and hydrogen-bonded silanol peaks**.** The intensity ratio of the peaks corresponding to hydrogen-bonded (vicinal) silanols (~ 3660 cm^{-1}) to that of isolated silanols (~ 3750 cm^{-1}) provided a measure of the relative population of the two types of silanol environments on the silica surface. When combined with the other Q-species fractions obtained from ^{29}Si NMR analysis, the FTIR results enabled the complete specification of surface silanol groups on the silica nanoparticles.

viii. Surface charge density estimation of silica nanoparticles

pH titrations of Ludox SM, Ludox HS, and Ludox TMA silica nanoparticle dispersions were performed using an automatic Orion Star T-910 titrator equipped with a calibrated pH electrode and an automated titrant delivery system. Prior to titration, the dispersions were dialyzed and

degassed, diluted with HPLC-grade water to a concentration of 6 wt.%, adjusted to an initial pH of ~10 using 0.1 M NaOH and allowed to equilibrate under continuous stirring to ensure uniform dispersion of the nanoparticles. The titrations were subsequently performed by controlled addition of 0.1 M HCl as the titrant while continuously monitoring the pH of the dispersion. The titrant was delivered automatically by the titrator in small incremental additions, and the pH response of the dispersions was recorded after equilibration following each addition. The titration was continued until the desired lower pH range (~ 2.5) was reached, generating a pH-titrant volume profile for the silica dispersions. A blank titration was also performed on HPLC water under identical conditions, and the two titration profiles were compared to estimate the surface charge density of the nanoparticles[3] as discussed in Note S7.

ix. Inductively coupled plasma optical emission spectroscopy (ICP-OES) measurements

Inductively coupled plasma optical emission spectroscopy (ICP-OES) measurements were performed to determine the presence of residual counter-ions in dispersions of dialyzed Ludox silica NPs. Prior to analysis, the nanoparticle dispersions were adjusted to pH ~ 2.5, corresponding to the isoelectric point (IEP) of silica, where electrostatic interactions between the particle surface and counter-ions are minimized. Under these conditions, any ions previously associated with the particle surface are expected to be released into the bulk solution. To isolate the dissolved ions, the dispersions were centrifuged to sediment the nanoparticles, and the supernatant was carefully collected for analysis. The collected supernatants were analyzed using a Perkin Elmer Optima 8300 Dual View ICP-OES equipped with argon-purged optics, and a calibration blank was prepared using nitric acid diluted with deionized water. The elemental concentrations were determined by comparing the measured emission intensities of the samples with those obtained from the calibration standards. This approach enabled the detection and quantification of any counter-ions remaining in the supernatant after dialysis of nanoparticle dispersions, thereby providing a direct assessment of the ionic species present in the bulk solution (Fig S9).

2. Supplementary Notes

Supplementary Note S1

Estimation of cumulant size and size distribution of nanoparticles from DLS measurements

The intensity weighted autocorrelation function, $g^2(\tau) - 1$ obtained from DLS measurements was fitted to a stretched exponential function, $\mathrm{A}_o \exp(-\frac{\tau}{\tau_{sc}})^{\beta_o}$, where τ is the lag time in seconds, and τ_{sc} and β_o are the free fit parameters. In our experimental data we observe that $g^2(\tau) - 1$ approaches a defined value, as $\tau \rightarrow 1$, and $g^2(\tau) - 1$ asymptotically converges to 0 for infinitely long lag times. This type of data has been previously analyzed by using a specific type of stretched exponential function, known as the Kohlrausch-William-Watts equation[4, 5] and has been used as a universal tool for studying various physical and chemical processes[6]. Specifically, in the case of light scattering[7], it has been used to study the kinetics of transformation processes like particle aggregation or gelation[8]. Here τ_{sc} and β_o are free-fit parameters which denote the following

β_o is a heterogeneity or polydispersity index of the relaxation process. Its magnitude ranges from 0 to 1 and is inversely related to the width of the distribution of the relaxation times. This implies that $\beta_o \rightarrow 1$ denotes homogeneity or monodisperse population, whereas $\beta_o \rightarrow 0$ denotes increase in polydispersity or increase in population of aggregates.

τ_{sc} is another free-fit parameter that is related to τ_s as $\tau_s = \frac{\tau_{sc}}{\beta_o} / \boldsymbol{\Gamma} \frac{\mathbf{1}}{\beta_o}$, where $\boldsymbol{\Gamma} \frac{\mathbf{1}}{\beta_o}$ is the gamma function of the inverse of the polydispersity index β_o. τ_s represents the magnitude of the relaxation rate at which the decay of the exponential function occurs and is thus unique for every curve. The relaxation rate τ_s is further related to the diffusivity D_{avg}, as $\tau_s = 1/D_{avg}q^2$, where q is the wave vector that is defined as $q = (4\pi n_o/\lambda) \sin\left(\frac{\theta}{2}\right)$. In our case, we use the refractive index n_o of the sample as 1.33, radiation wavelength of the laser, λ as 658 nm, and the scattering angle θ as 90°. The diffusivity, D_{avg} for a particular curve is the average diffusivity of the particles of various populations (singlets, doublets, and larger cluster aggregates) present in the sample. D_{avg} is related to the corresponding average hydrodynamic radius, Rh_{avg} by the Stokes-Einstein's relation as $Rh_{avg} = k\mathrm{T}/6\pi\eta D_{avg}$, where k is the Boltzmann constant, T is the temperature of the sample and η is the viscosity of water.[4] Assuming every species detected is a sphere, we determine the particle size as twice the Rh_{avg}.

Supplementary Note S2

Estimation of dispersion salinity (background salt concentration) from conductivity experiments

Conductivity of the dispersions, S was experimentally measured as a function of pH with a conductivity probe (Oakton CON 550) that was pre-calibrated with standards 84 µS/cm, 1413 µS/cm, and 12.88 mS/cm and 111.8 mS/cm. The background salt concentration or dispersion salinity, c was determined from the empirical relation[9] $c = a_o S,$ where a_o is a theoretical constant that can be estimated for water in the presence of certain ions, (Na^+ and Cl^- here), and is given by the expression $a_o = 4\pi\, \eta\, a_h / e^2 N_A$ where η is the viscosity of water at 25℃, e is the electronic charge, N_A is the Avogadro number, and a_h is the average hydrodynamic radii of Na^+ and Cl^- ions, which is 1.01 Å, and 1.82 Å, respectively. The variation of conductivity, S as measured experimentally and estimated salt concentration, c of 9 nm silica nanoparticles with pH is as shown below (Note S2 Fig. 1)

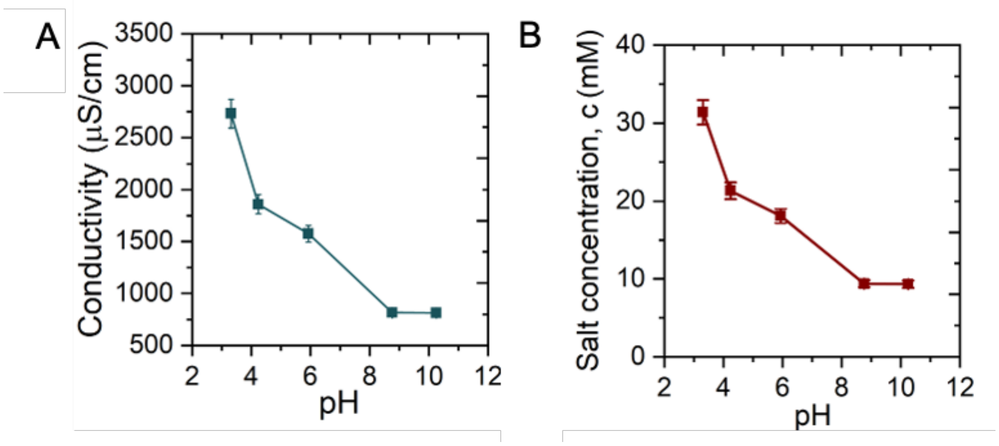

Note S2 Fig 1. Estimating dispersion salinity from conductivity experiments. a) Electrical conductivity of 9 nm (Ludox SM silica nanoparticles) measured experimentally as a function of pH. Points are experimental data and error bars are from uncertainties of conductivity measurements. b) Background salt concentration of 9 nm silica nanoparticles across the pH range as estimated from the conductivity measurements.

Supplementary Note S3

Estimating U(r) vs r from analysis of SAXS data

Small-angle X-ray scattering (SAXS) measurements were performed on aqueous dispersions of silica nanoparticles with mean diameters of 9 nm, 15 nm, and 27 nm after 90 days of aging, and the measured scattering intensity I(q) was analyzed using the standard decoupling approximation $I(q) = P(q)S(q)$, where the particle form factor P(q) was first independently determined from SAXS using a hard-sphere model. The particle radii extracted from the form-factor analysis were subsequently used as fixed inputs for all structure-factor calculations. The black curves shown in Fig. 2 A-C of the manuscript correspond to structure factors calculated using the Hayter-Penfold mean spherical approximation (MSA) model[10] implemented in SasView software and are not fits to the experimental data, instead, they are fully determined from independently measured experimental parameters. Specifically, the particle radius was fixed from SAXS form-factor analysis, the volume fraction was fixed at ϕ = 0.06 based on sample preparation, the particle charge Q_P was entered directly as an input parameter estimated from surface charge density measurements, the ionic strength was fixed using conductivity measurements, and the dielectric constant of water was fixed at 71.08 for all datasets, with no parameters allowed to vary. As a result, the one-Yukawa Hayter-Penfold structure factors represent the expected scattering response arising solely from electrical double-layer repulsion as described by classical DLVO theory. To extend this analysis, the repulsive component of the DLVO interaction was mapped onto a Yukawa potential by explicitly assuming the linearized Poisson-Boltzmann (Debye-Hückel) approximation, with the screening parameter defined as $Z_1 = 2a\kappa$, where κ^{-1} is the Debye length determined from the experimentally measured ionic strength, and the Yukawa amplitude given by $K_1 = 2\pi\varepsilon\varepsilon_o a{\psi_o}^2\beta$, where zeta potential was used as the effective surface potential, ψ_o, and the geometric factor, β was fixed to 1 for all calculations. These parameters were calculated independently for each particle size and were fixed in subsequent modeling. The red curves shown in Figure 2A-C of manuscript were obtained using the Two-Yukawa structure factor model[11] in SasView, in which the total interaction potential is expressed as the sum of a repulsive and an attractive Yukawa term; in these fits, the repulsive parameters Z_1 and K_1 were fixed to the DLVO-derived values for each particle size, while the attractive parameters Z_2 and K_2 were treated as free fitting parameters. The additional attractive Yukawa contribution represents an effective short-range attraction capturing van der Waals interactions together with non-DLVO effects such as charge-fluctuation mediated forces in our study, and the comparison between the fully constrained one-

Yukawa calculations and the minimally extended two-Yukawa fits provides a physically transparent framework to quantify deviations from classical DLVO behavior across particle sizes.

For the 27 nm particles, the pair interaction potential $U(r)$ was obtained from the screened Coulomb potential used in the Hayter-Penfold MSA model[10], $U(r) = 4\pi\varepsilon\varepsilon_0 a^2 \psi_0^2 \exp\,[-\kappa(r - 2a)]/r$ for $r > 2a$. For the 15 nm and 9 nm particles, the interaction potential was represented using a two-Yukawa form[11], $U(x)/k\mathrm{T} = K_1 e^{-Z_1(x-1)}/x + K_2 e^{-Z_2(x-1)}/x$, corresponding to the repulsive and attractive contributions used in the Two-Yukawa structure factor model.

Supplementary Note S4

Theoretical calculation of DLVO interaction energy

The total DLVO interaction energy, U_{DLVO}, between two identical spherical colloidal particles of radius a separated by a center-to-center distance, r, was estimated as the sum of electrostatic double-layer repulsion and van der Waals attraction. The electrostatic contribution was calculated using the classical framework developed by Verwey and Overbeek, based on solutions of the linearized Poisson-Boltzmann (PB) equation. The electrostatic interaction energy can be written as[12]

$$U_{\mathrm{EDL}}(x) = \frac{2\pi\varepsilon\varepsilon_0 a\psi_0^2\,\beta e^{-2a\kappa(x-1)}}{x} \quad (1)$$

where $\varepsilon\varepsilon_0$ is the dielectric permittivity of the solvent, ψ_0 is the surface potential, here taken to be equal to the experimentally determined zeta potential (See note S6 for more details), and $\kappa^{-1}$is the Debye screening length. The dimensionless separation parameter, $x = r/2a$ corresponds to the center-to-center distance, r scaled by the particle diameter. The function β is a geometric correction factor that accounts for finite particle size and curvature effects and is given by a rational polynomial expression derived by Verwey and Overbeek. For simplicity, and to retain analytical transparency, we assume $\beta = 1$, which corresponds to neglecting curvature-induced corrections to the electrostatic interaction. This approximation is appropriate when the dominant separation dependence is governed by the exponential screening term $e^{-\kappa x}$ and when the goal is to estimate the magnitude and range of electrostatic repulsion rather than capture higher-order geometric effects. Under this assumption, the electrostatic interaction energy simplifies to

$$U_{\mathrm{EDL}}(x) = \frac{2\pi\varepsilon\varepsilon_0 a\psi_0^2 e^{-2a\kappa(x-1)}}{x} \quad (2)$$

The van der Waals attractive interaction between the two spheres was calculated using the exact pairwise summation result for identical spheres. The interaction energy is given by

$$U_{\mathrm{vdW}}(x) = -\frac{A}{6}\left[\frac{1}{2(x^2-1)} + \frac{1}{2x^2} + \ln\left(\frac{x^2-1}{x^2}\right)\right] \quad (3)$$

A is the Hamaker constant, taken as[13] 4.6×10^{-21} J. The total DLVO interaction energy is then obtained as

$$U_{\mathrm{DLVO}}(x) = U_{\mathrm{EDL}}(x) + U_{\mathrm{vdW}}(x) \quad (4)$$

Supplementary Note S5

Justification for Surface Potential being equivalent to the experimentally measured zeta potential

ζ potential represents an electrical potential from electrokinetic experiments such as electrophoresis and streaming potential, associated with tangential displacements of the liquid along the particle wall. It is known to measure the electrical potential characterizing the diffuse part of the double layer considering the Stern's picture for the electrical double layer[14]. Since the diffuse layer potential is a key measure for a double-layer overlap originating the electrostatic repulsion between objects, the ζ potential can be reasonably utilized for describing aggregation and colloidal stability. In fact, Verwey and Overbeek noted that the ζ potential is likely to be relatively close to the diffuse layer potential, compared to the surface potential.[15] More importantly, force measurements using surface force apparatus for two mica surfaces by Pashley[16] confirmed that an effective surface potential obtained from the measurements and DLVO theory (i.e., the one to be used for interaction potentials between objects) corresponds to an electrical potential at the outer Helmholtz plane (i.e., the diffuse layer potential) rather than the surface potential obtained from the surface charge density [16, 17].

Supplementary Note S6

Surface chemistry of silica nanoparticles. Estimating fraction of various silanol groups on silica nanoparticles using NMR and FTIR spectroscopy

The surface chemistry of Ludox silica nanoparticles (9, 15, and 27 nm) was quantified by combining ^{29}Si CP-MAS NMR and FTIR spectroscopy. The NMR spectra provide the relative fractions of Q^2, Q^3, and Q^4 silicon environments on the silica surface, while FTIR spectroscopy allows further partitioning of the Q^3 population into isolated and vicinal silanol groups. By integrating information from both techniques, the fractions of all surface species Q^2 (geminal silanols), Q^3 isolated silanols, Q^3 vicinal silanols, and Q^4 siloxane groups were estimated for each nanoparticle size.

a. Determination of Q^2, Q^3, and Q^4 fractions from ^{29}Si CP-MAS NMR spectra

The ^{29}Si CP–MAS NMR spectra of silica nanoparticles were analyzed to quantify the relative abundance of Q^n silicon environments on the particle surface. In silica systems, the Q^n notation describes a silicon atom bonded to n bridging oxygen atoms (Si–O–Si) and 4n hydroxyl groups. The characteristic resonances for hydroxylated silica occur at approximately −89 ppm, −99 ppm, and −109 ppm, corresponding to Q^2, Q^3, and Q^4 environments, respectively[1, 18]. For each nanoparticle size, the experimental NMR spectrum was deconvoluted into three Gaussian components centered at these characteristic chemical shifts (Note S6 Fig. 1). After baseline correction, the total spectral envelope was fit using constrained Gaussian functions corresponding to Q^2, Q^3, and Q^4 contributions[19]. The area under each fitted peak was then calculated to determine the relative populations of the three species. The fractional abundance of each Q-species was obtained by normalizing the peak areas with respect to the total integrated intensity of the spectrum. These NMR-derived fractions provide the overall distribution of Q^2, Q^3, and Q^4 on the nanoparticle surface.

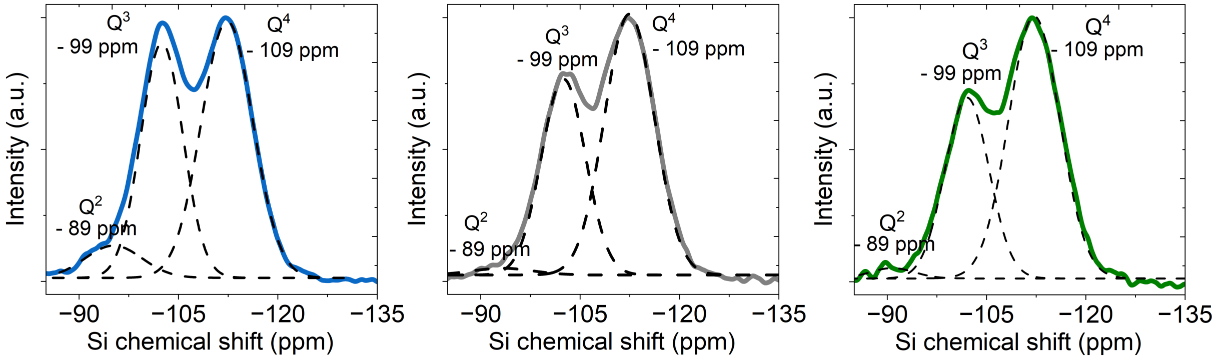

Note S6 Fig. 1. ^{29}Si CP-MAS NMR spectra of Ludox silica nanoparticles with diameters of 9, 15, and 27 nm. The spectra were deconvoluted into Gaussian components corresponding to Q^2 (−89 ppm), Q^3 (−99 ppm), and Q^4 (−109 ppm) silicon environments.

b. Separation of isolated and vicinal silanols from FTIR spectra

While NMR provides the total fraction of Q^3 silanol groups, it does not distinguish between isolated and hydrogen-bonded silanols. This distinction was obtained using FTIR spectroscopy of the O–H stretching region. Silica surfaces exhibit characteristic infrared bands associated with different silanol environments. Isolated silanol groups produce a sharp absorption near 3750 cm^{-1}, whereas hydrogen-bonded or vicinal silanol groups appear at lower wavenumbers near 3660 cm^{-1}. FTIR spectra were collected for the three silica nanoparticle sizes and the relative intensities of the isolated and vicinal silanol bands were analyzed (Note S6 Fig. S2). The peak intensities at the characteristic wavenumbers were used to determine the relative contributions of the two silanol types. The ratio of the intensities of the vicinal and isolated bands provided a measure of the relative population of the vicinal and isolated groups on the silica surface.

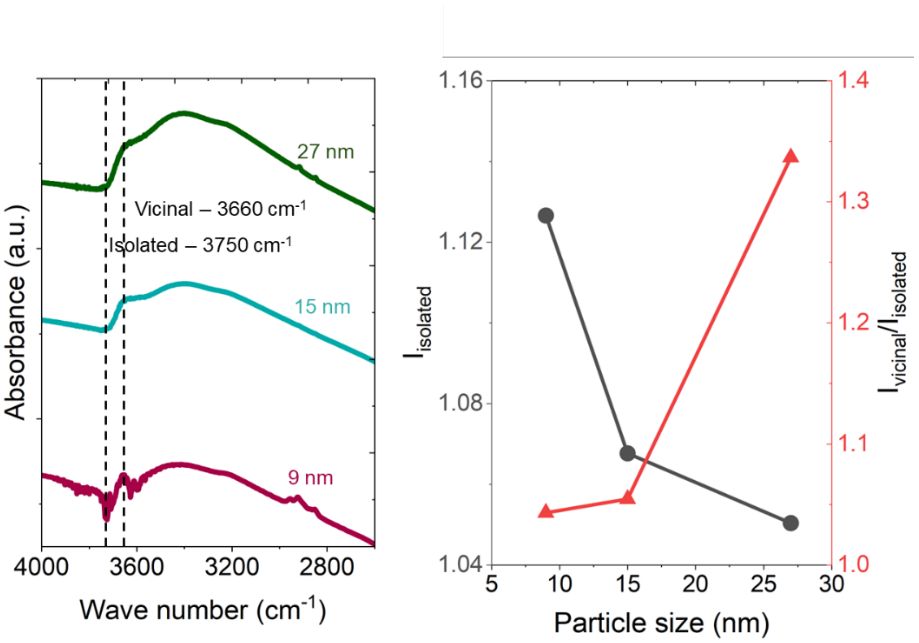

Note S6 Fig. 2. FTIR spectra of silica nanoparticles in the O–H stretching region highlighting bands associated with different silanol environments. The sharp peak near 3750 cm^{-1} corresponds to isolated silanol groups, while the broader band near 3660 cm^{-1} corresponds to vicinal silanols. The relative intensities of these bands were used to determine the fraction of isolated and vicinal silanols within the total Q^3 population.

c. Combined NMR–FTIR analysis for complete surface silanol speciation

The final fractions of each surface species were obtained by combining the information derived from NMR and FTIR measurements. The NMR analysis yields the overall fractions of Q^2, Q^3, and Q^4 species present on the silica surface. Since Q^3 sites correspond to single silanol groups, the FTIR intensity ratios were used to further partition this Q^3 fraction into isolated and vicinal silanol contributions. Specifically, the fraction of isolated silanols within the Q^3 population was calculated from the ratio of the isolated silanol peak intensity to the sum of isolated and vicinal intensities. The remaining portion of the Q^3 fraction was assigned to vicinal silanols. Multiplying these FTIR-derived percentages with the NMR-derived Q^3 fraction yields the final fractions of isolated and vicinal silanols on the silica surface. The Q^2 fraction obtained from NMR corresponds to geminal silanol groups, while the Q^4 fraction represents fully condensed siloxane sites. Using this combined approach, the surface silanol distribution for each nanoparticle size was determined as the fractions of geminal silanols (Q^2), isolated silanols (Q^3 isolated), vicinal silanols (Q^3 vicinal), and siloxane groups (Q^4), as summarized in Fig. S6. This integrated NMR-

FTIR methodology thus provides a consistent framework to quantify the surface chemistry of silica nanoparticles and its dependence on particle size (Note S6 Figure S3)

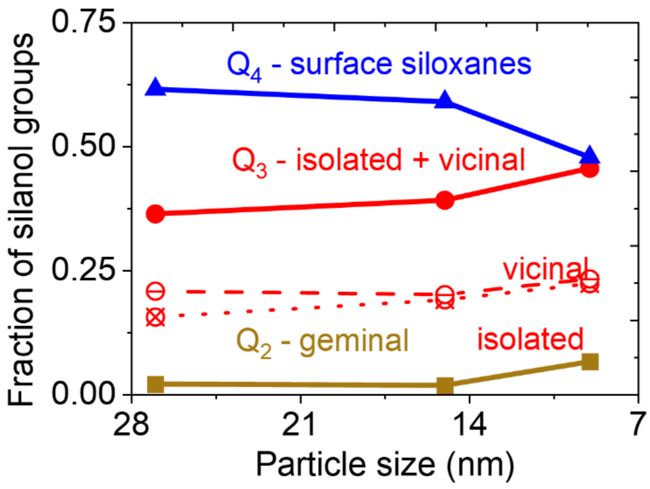

Note S6 Figure S3. Combined NMR–FTIR analysis showing the calculated fractions of surface species on silica nanoparticles of different sizes. The distribution includes geminal silanols (Q^2), isolated silanols (Q^3), vicinal silanols (Q^3), and fully condensed siloxane groups (Q^4), obtained by partitioning the NMR-derived Q^3 fraction using FTIR intensity ratios.

Supplementary Note S7

Experimental determination of mean pK_a of silica nanoparticles and other NP systems

The intrinsic acidity constant of silanol groups on silica surfaces was estimated following the procedure proposed by Milonjić for oxide-electrolyte interfaces[20]. At pH values above the point of zero charge, surface ionization of silanol groups proceeds according to $\mathrm{SiOH} \rightleftharpoons \mathrm{SiO^-} + \mathrm{H^+}$ (See note S7 for details). The apparent surface acidity quotient pQ_acan be written as

	$pQ_a = p\mathrm{H} - \log\left(\frac{\alpha}{1-\alpha}\right)$	(5)

where α represents the fraction of dissociated surface groups relative to the total number of ionizable surface sites. According to the site-binding framework, the measured pQ_a depends not only on the intrinsic acidity of surface sites but also on the electrostatic potential generated by surface charge. As the degree of ionization increases, electrostatic repulsion between negatively charged sites raises the apparent acidity constant. Consequently, the experimentally observed pQ_a values increase with increasing surface ionization.

To isolate the intrinsic constant, Milonjić proposed evaluating the dependence of pQ_aon the degree of ionization and extrapolating to the limit of zero surface charge. In this limit, $\alpha \to 0$ and the surface potential vanishes. Under these conditions, electrostatic interactions are absent and the apparent acidity constant equals the intrinsic surface ionization constant, $pQ_a(\alpha \to 0) = pK_{a2}^{int}$. Thus, pK_{a2}^{int} can be obtained by plotting pQ_a as a function of the degree of ionization α (or equivalently 10α as commonly used for graphical scaling) and extrapolating the trend to $\alpha = 0$. The intercept of this extrapolation corresponds to the intrinsic acidity constant of the surface sites. In this work, pQ_avalues were calculated from experimental titration data using the expression above. The resulting pQ_avalues were plotted against 10α, and a linear regression was performed in the low-ionization regime where electrostatic effects are approximately linear with respect to surface charge. Extrapolation of the fitted line to $10\alpha = 0$ yielded, $pK_{a2}^{int} \sim 8.3$ for all 9 nm, 15 nm, 27 nm silica nanoparticles, which is consistent with previously reported values[21, 22] for silica surfaces.

The linear extrapolation was performed using data in the pH range of approximately 6-10. This region corresponds to the onset of silanol deprotonation where the degree of surface ionization remains relatively small ($\alpha \ll 1$). Under these conditions, electrostatic interactions between neighboring charged sites are weak and the relationship between the apparent acidity quotient pQ_a and the degree of ionization is approximately linear. At lower pH values, the surface charge

approaches zero and uncertainties in the determination of small charge densities can introduce significant experimental error. At higher pH values, the surface becomes increasingly charged, leading to stronger electrostatic repulsion between deprotonated sites and deviations from linear behavior. Furthermore, dissolution of silica becomes significant above pH ≈ 10, which can alter the surface site density and compromise the reliability of the titration data. Therefore, restricting the analysis to the intermediate pH range ensures that the extrapolation to $\alpha \rightarrow 0$ provides a robust estimate of the intrinsic surface acidity constant.

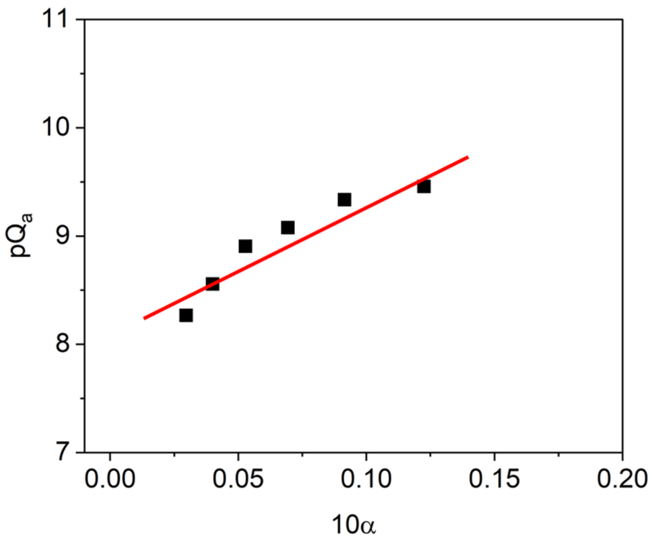

Note S7 Fig S1 Determination of the intrinsic silanol acidity constant using the Milonjić method. The apparent acidity quotient $pQ_a = p\mathrm{H} - \log\left(\frac{\alpha}{1-\alpha}\right)$ is plotted as a function of 10α, where α is the fraction of dissociated surface sites. A linear fit to the low-ionization regime ($\alpha \ll 1$) is extrapolated to $10\alpha = 0$, corresponding to zero surface charge. The intercept yields the intrinsic constant $pK_a^{\mathrm{int}} \approx 8.3$.

Supplementary Note S8

Estimation of surface charge density of silica nanoparticles from pH titrations

Silica nanoparticle dispersions (Ludox SM-30, HS-40, and TMA) were purified and prepared following the procedure described in the Methods section. Two independent potentiometric titrations were conducted under identical experimental conditions:
(i) a blank titration consisting of HPLC water without particles, and
(ii) a dispersion titration containing a known concentration of silica nanoparticles in water. Both titrations were carried out by incremental addition of 0.1 M HCl as the titrant. The pH was monitored using a calibrated glass electrode (Hannah Instruments HI 4112), with calibration performed using standard buffer solutions (pH 4, 7, and 10) prior to each experiment.

At a given pH, the net amount of charge determining ions associated with the silica surface was obtained from the difference between the amount of HCl required to reach that pH in the dispersion and in the blank system. Since the titrations were initiated from basic conditions, this differential quantity corresponds to the net uptake of protons by deprotonated silanol groups on the silica surface, while accounting for proton consumption in the bulk solution as reported by previous studies in the literature[3, 20]. The surface charge density (σ), was calculated by converting the net proton uptake to charge per unit surface area using the experimentally measured specific surface area of the silica particles by the expression $\sigma = PF/a_{sp}$, where P is the net amount of protons adsorbed per unit mass of silica (mol/g), obtained from the differential titration, F is the Faraday constant, and a_{sp} is the specific surface area of the silica particles (m^2/g) as determined from experimental specific surface area measurements. The pH titration curves of 9 nm silica nanoparticle dispersion and the blank titration are shown in Note S5 Fig. S1. The surface charge density obtained from the potentiometric titrations was further used to estimate the net charge per particle. Assuming spherical particles, the total charge per particle, Q_P was calculated from the relation $Q_P = \sigma \times 4\pi a^2$, where a is the particle radius.

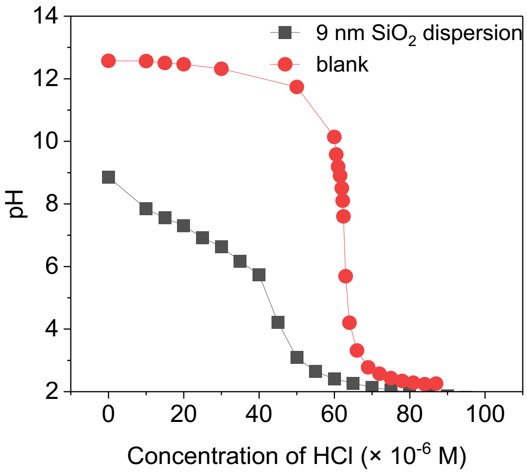

Note S8 Figure S1. (A) Potentiometric titration curves showing pH as a function of added HCl for a 9 nm silica nanoparticle dispersion and the corresponding blank titration performed in HPLC water. The difference between the two curves provides the net proton uptake associated with surface silanol groups on the silica nanoparticles.

Supplementary Note S9

Free energy of dissociation of surface silanol groups

The free energy associated with the dissociation of surface silanol groups was estimated using standard thermodynamic relations for acid–base equilibria. The deprotonation of silanol groups on the silica surface can be written as[23, 24]

$$\mathrm{SiOH} \rightleftharpoons \mathrm{SiO^-} + \mathrm{H^+} \tag{6}$$

where a surface silanol group releases a proton into the surrounding aqueous solution. The thermodynamic driving force for this dissociation can be expressed in terms of the reaction free energy. From the general relation between free energy and the reaction quotient, the free energy change of the reaction is given by[25]

$$\Delta G = \Delta G^\circ + RT\ln\, Q \tag{7}$$

where R is the universal gas constant T is the absolute temperature, and Q is the reaction quotient. For the silanol dissociation reaction, the reaction quotient is written as

$$Q = \frac{[a_{\mathrm{SiO^-}}][a_{\mathrm{H^+}}]}{[a_{\mathrm{SiOH}}]} \tag{8}$$

where a_i represents the activity of species i. The standard free energy change is related to the acid dissociation constant K_a through

$$\Delta G^\circ = -RT\ln\, K_a \tag{9}$$

Substituting (4) in (2) yields to

$$\Delta G = RT\ln\left(\frac{Q}{K_a}\right) \tag{10}$$

which, after substituting the reaction quotient, becomes

$$\Delta G = RT\ln\left(\frac{[a_{\mathrm{SiO^-}}][a_{\mathrm{H^+}}]}{[a_{\mathrm{SiOH}}]K_a}\right) \tag{11}$$

Expanding the logarithmic terms gives

	$$\Delta G = RT\ln\left(\frac{a_{\mathrm{SiO^-}}}{a_{\mathrm{SiOH}}}\right) + RT\ln\left(a_{\mathrm{H^+}}\right) - RT\ln\left(K_a\right)$$	(12)

Using the relation $pH = -\log_{10}(a_{\mathrm{H^+}})$ and $pK_a = -\log_{10}(K_a)$, along with the conversion between natural and base-10 logarithms ($RT\ln\ x = 2.303RT\log_{10} x$), (equation 7) can be written as

	$$\Delta G = 2.303RT\left(pK_a - pH + \log_{10}\left(\frac{[a_{\mathrm{SiO^-}}]}{[a_{\mathrm{SiOH}}]}\right)\right)$$	(13)

This relation shows that the free energy of silanol dissociation depends on both the solution pH relative to the intrinsic acidity of the surface groups and the relative population of protonated and deprotonated surface sites. When the solution pH is significantly larger than the intrinsic pK_a of the silanol group, the free energy becomes negative and dissociation is thermodynamically favored, leading to an increased fraction of negatively charged $\mathrm{SiO^-}$ groups. Conversely, at pH values well below the intrinsic pK_a, protonated silanol groups SiOH are energetically favored. At $pH = pK_a$, the free energy contribution from the solution environment is balanced, and the dissociation state is determined by the relative population of surface species.

3. Supplementary Figures

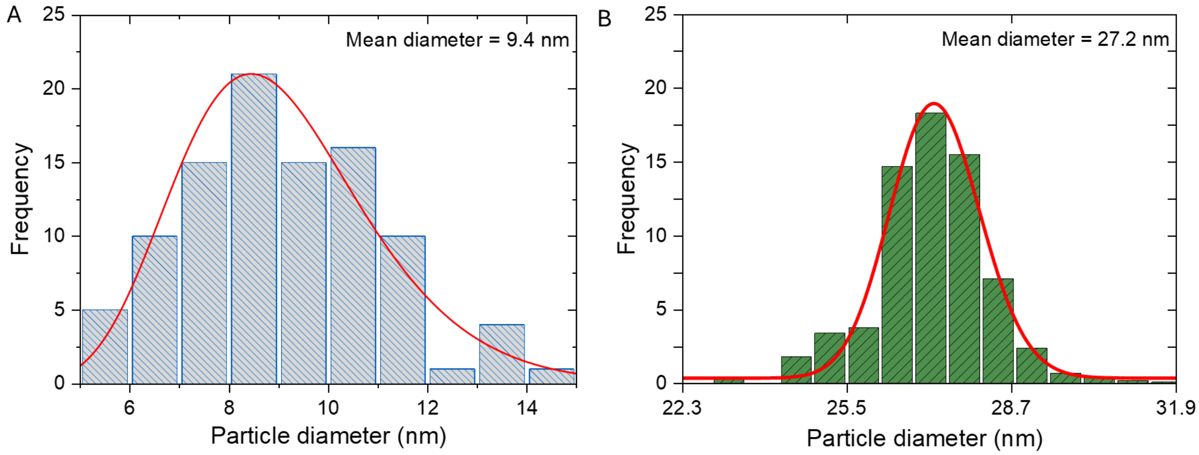

Fig S1. Size distribution of 9 nm and 27 nm silica nanoparticles at pH 8.7 (Day 90) from cryo-TEM images.

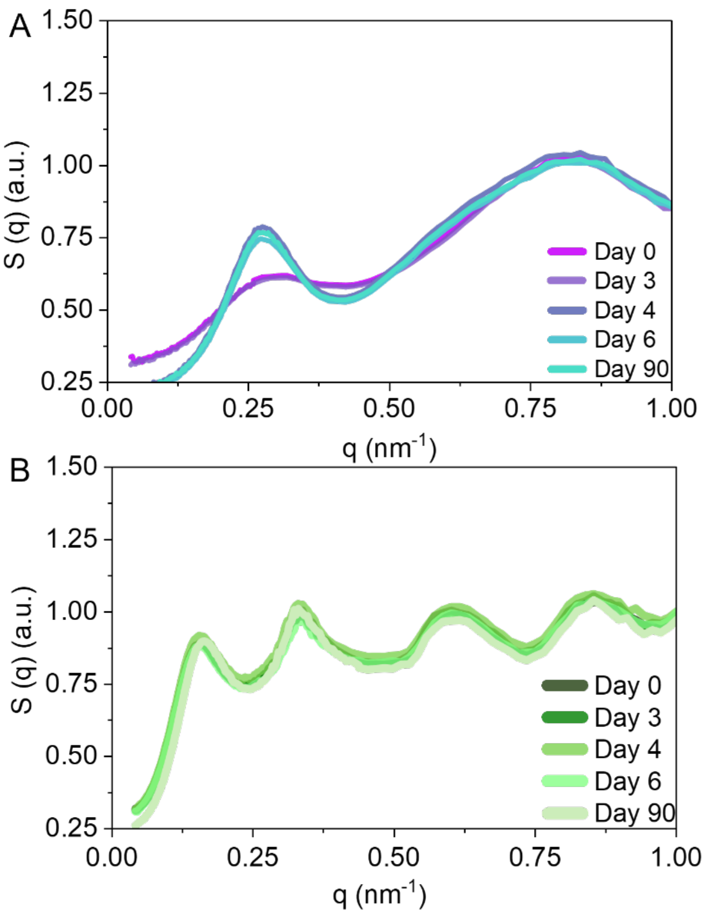

Fig. S2. Time evolution of structure factors extracted from SAXS measurements. (A) Structure factor S(q) of 9 nm silica nanoparticles measured at different aging times. (B) Structure factor S(q) of 27 nm silica nanoparticles measured at corresponding times, showing the emergence of structural correlations during aging.

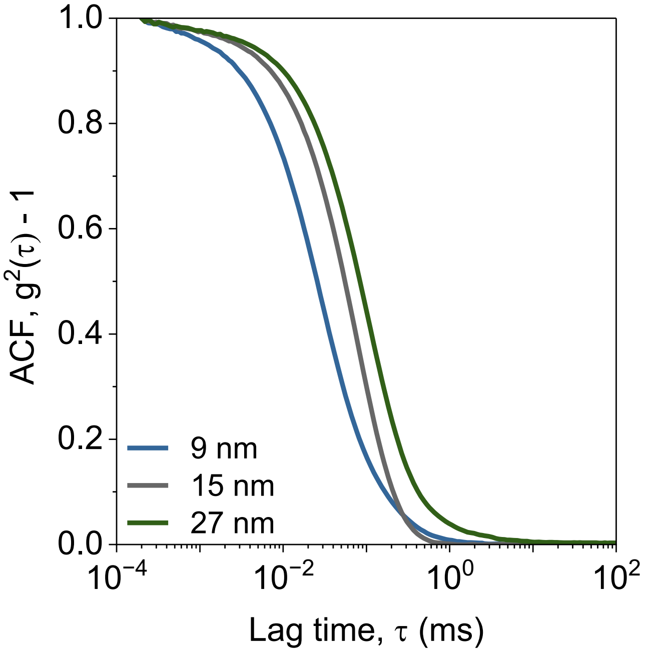

Fig S3. ACF vs lag time of 9 nm, 15 nm, and 27 nm silica nanoparticles showing dispersed state of the dispersion at day 0.

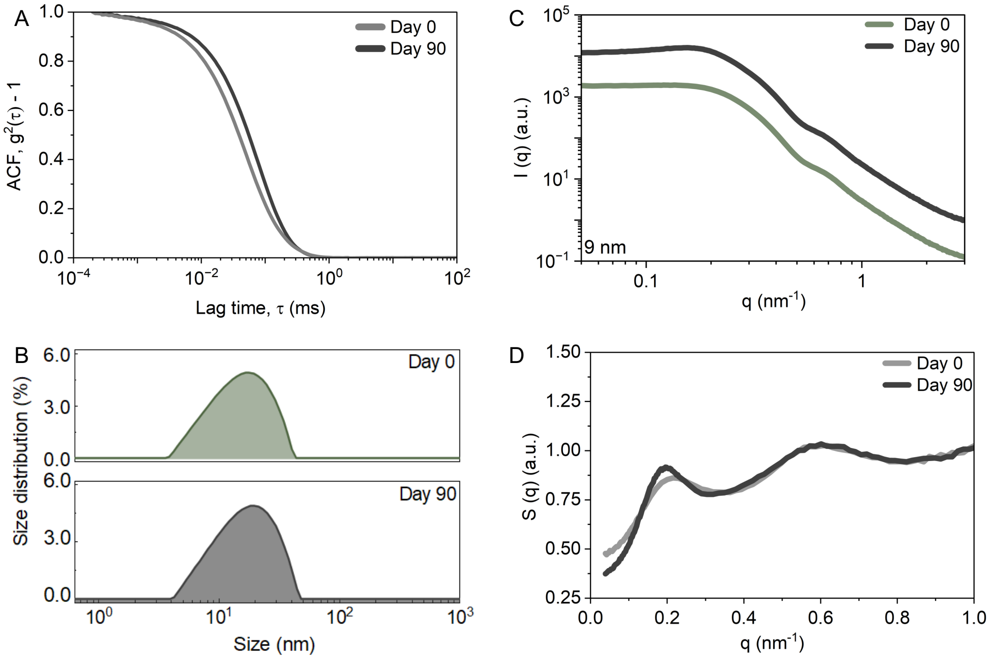

Fig. S4. (A-B) DLS autocorrelation functions and size distributions of 15 nm silica NPs at Day 0 and Day 90, showing a minor increase in particle size by ~2 nm after 90 day equiliberation, which is unlike the significant increase in size observed in smallest NPs. (C-D) Experimental scattering intensity I(q) and corresponding structure factor S(q) extracted at Day 0 and Day 90 from SAXS, showing the transition from dispersed particles to weak structural correlations at Day 90.

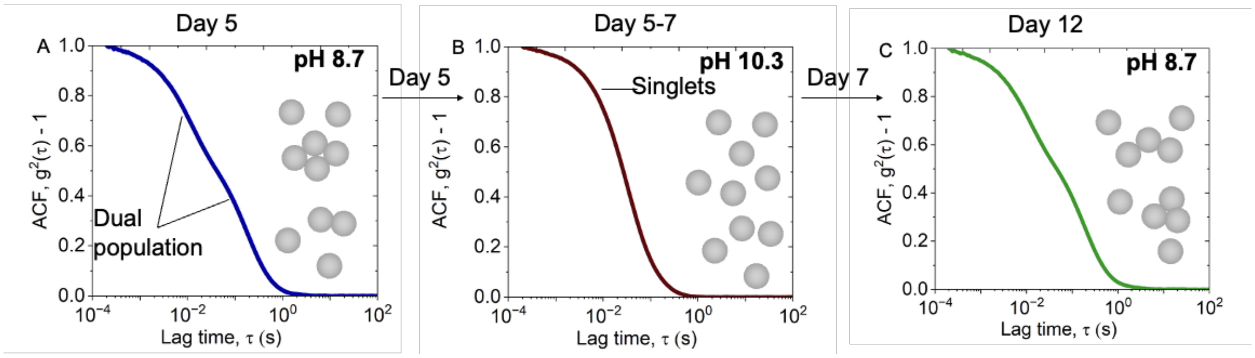

Fig. S5. Reversibility of nanoparticle cluster formation for 9 nm silica nanoparticles. Equilibrium silica clusters formed near the clustering pH 8.7 spontaneously redisperse upon increasing the pH to 10.3, demonstrating that the particle association is governed by reversible physical interactions rather than irreversible chemical bonding.

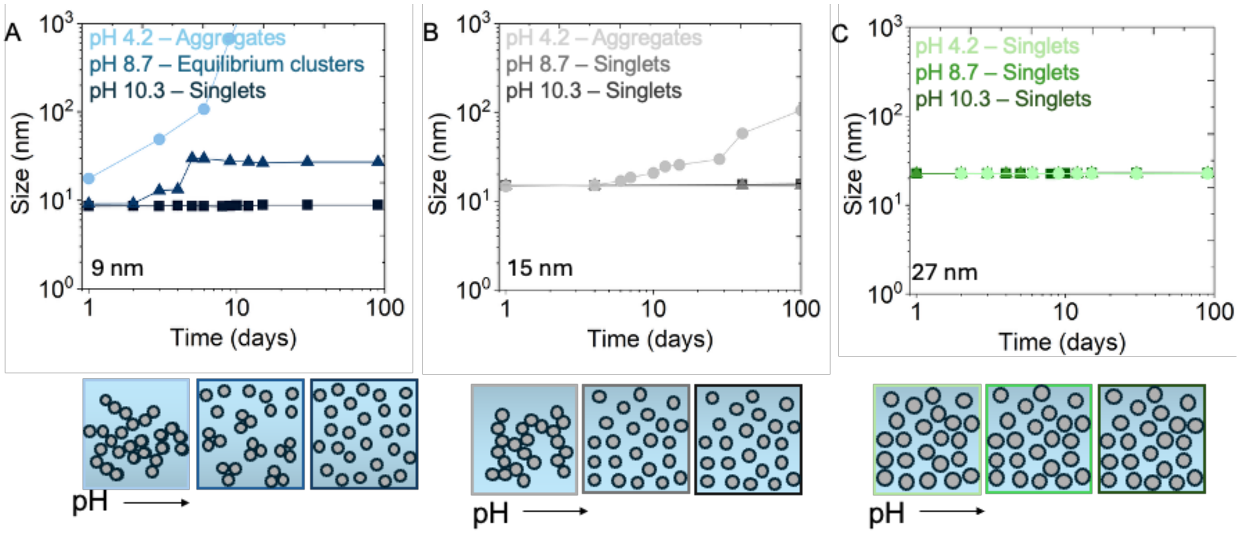

Fig. S6. Time-dependent cumulant particle size from DLS measurements for (A) 9 nm, (B) 15 nm, and (C) 27 nm silica nanoparticles across the pH range over ~100 days.

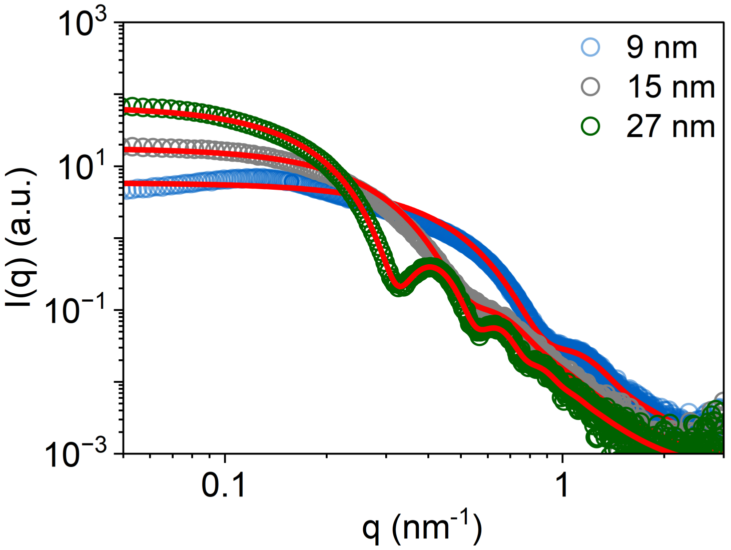

Fig. S7. Scattering intensity I(q) (open circles) with corresponding form-factor fits P(q) (red lines), of 1 wt% 9 nm, 15 nm and 27 nm silica nanoparticles as a function of time at pH 10 indicating dispersed particles.

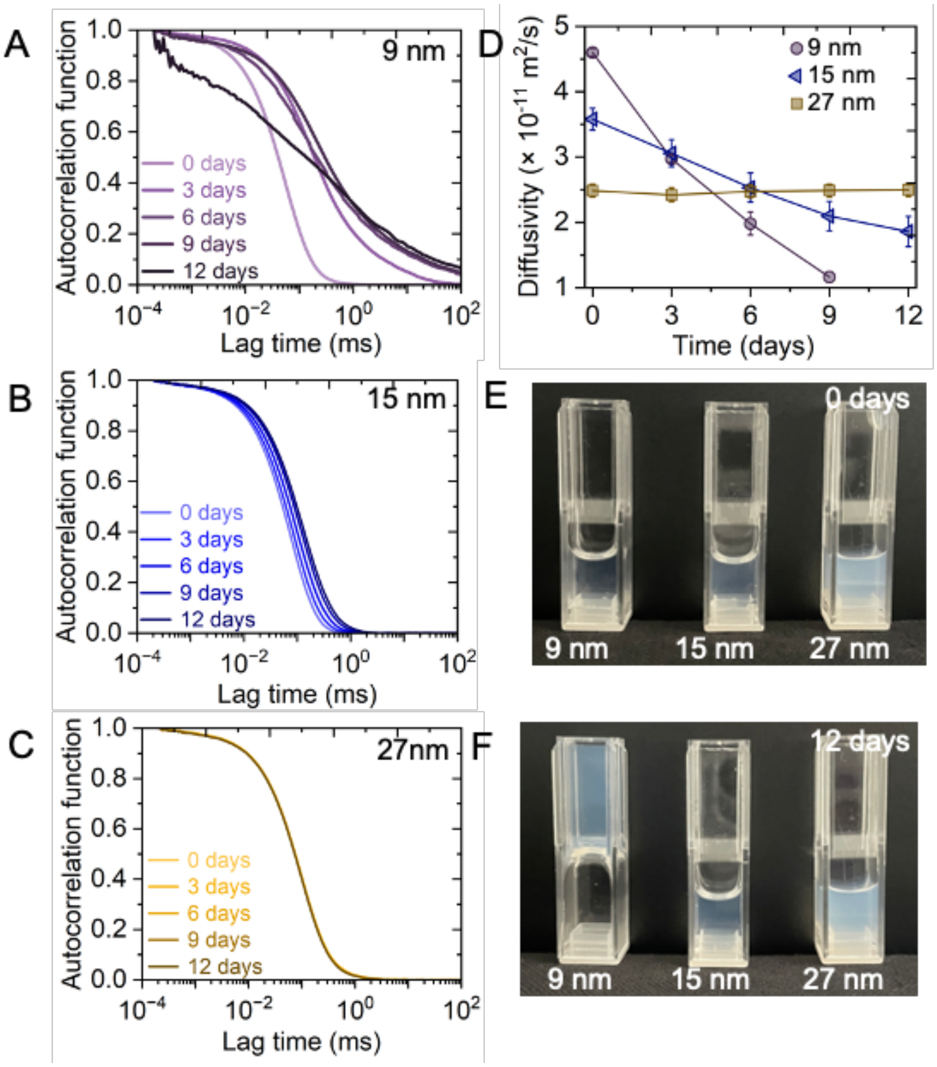

Fig. S8. Time-dependent DLS analysis of silica nanoparticle aggregation at pH ~ 5. (A–C) Autocorrelation functions for 9 nm, 15 nm, and 27 nm silica nanoparticles measured over time. (D) Diffusivity of the dominant particle population as a function of time for the three particle sizes. (E) Photographs of silica dispersions immediately after pH adjustment showing turbidity differences. (F) Photographs of the same samples after 12 days showing gel formation for the smallest particles.

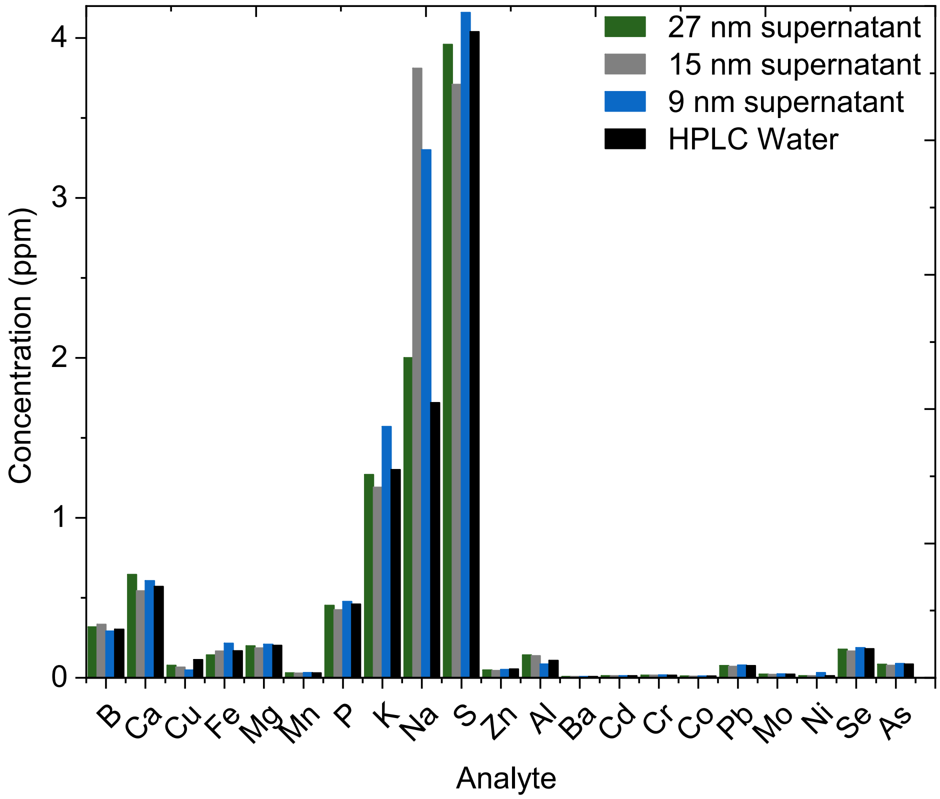

Fig. S9. ICP-OES analysis of ionic species present in the supernatant after dialysis of silica nanoparticle dispersions I. HPLC-grade wateer, confirming removal of residual counter-ions and verifying the ionic composition of the dispersion medium.

4. Supplementary Tables

Particle diameter (nm)	Ionic strength (M)	Charge per particle (e.u.)	Volume fraction
9.4	0.01	-114.5	0.06
15.4	0.01	-101.7	0.06
27.0	0.01	-150.5	0.06

Table S1: Input parametes for one-Yukawa structure factor described by the Hayter-Penfold mean spherical approximation for charged spheres (black curves in Fig. 2A-C). Dielectric constant of water and temeprature was kept fixed respectively at 71.08, and 298K for all three samples. All inputs given here were determined using independent measurements presented in the main article and SI.

Particle diameter (nm)	ζ potential (mV)	κ^{-1} (nm)	Z_1	K_1	Z_2	K_2
9.4	-31.3	3.1	3.0	5.1	15.4	-19.5
15.4	-45.1	3.1	5.0	18.8	6.8	-22.0
27.0	-60.0	3.4	-	-	-	-

Table S2. Input parameters used for SAXS modeling and interaction potential calculations using two-Yukawa potential (red curves in Fig. 2A-C). $Z_1 = 2a\kappa$, where κ^{-1} is the Debye length. $K_1 = 2\pi\varepsilon\varepsilon_o a\zeta^2\beta/kT$, where ζ potential was used as the effective surface potential. Z_1, K_1, Z_2, and K_2 are dimensionless.

References

(1) Chuang, I.-S.; Maciel, G. E. Probing hydrogen bonding and the local environment of silanols on silica surfaces via nuclear spin cross polarization dynamics. *Journal of the American Chemical Society* **1996**, *118* (2), 401-406.
(2) Kamiya, H.; Mitsui, M.; Takano, H.; Miyazawa, S. Influence of particle diameter on surface silanol structure, hydration forces, and aggregation behavior of alkoxide-derived silica particles. *Journal of the American Ceramic Society* **2000**, *83* (2), 287-293.
(3) Bolt, G. Determination of the charge density of silica sols. *The journal of physical chemistry* **1957**, *61* (9), 1166-1169.
(4) De Oliveira, V.; De Morais, W.; Pereira, M.; Fonseca, J. Dynamic light scattering in semidilute and concentrated chitosan solutions. *European polymer journal* **2012**, *48* (11), 1932-1939.
(5) Scopigno, T.; Yannopoulos, S.; Scarponi, F.; Andrikopoulos, K.; Fioretto, D.; Ruocco, G. Origin of the λ transition in liquid sulfur. *Physical review letters* **2007**, *99* (2), 025701.
(6) Iselau, F.; Xuan, T. P.; Trefalt, G.; Matic, A.; Holmberg, K.; Bordes, R. Formation and relaxation kinetics of starch–particle complexes. *Soft Matter* **2016**, *12* (47), 9509-9519.
(7) Shamblin, S. L.; Hancock, B. C.; Dupuis, Y.; Pikal, M. J. Interpretation of relaxation time constants for amorphous pharmaceutical systems. *Journal of pharmaceutical sciences* **2000**, *89* (3), 417-427.
(8) Narayanan, J.; Deotare, V. W.; Bandyopadhyay, R.; Sood, A. Gelation of aqueous pectin solutions: A dynamic light scattering study. *Journal of colloid and interface science* **2002**, *245* (2), 267-273.
(9) Wang, S.; Walker-Gibbons, R.; Watkins, B.; Flynn, M.; Krishnan, M. A charge-dependent long-ranged force drives tailored assembly of matter in solution. *Nature Nanotechnology* **2024**, *19* (4), 485-493.
(10) Hayter, J. B.; Penfold, J. An analytic structure factor for macroion solutions. *Molecular Physics* **1981**, *42* (1), 109-118.
(11) Liu, Y.; Chen, W.-R.; Chen, S.-H. Cluster formation in two-Yukawa fluids. *The Journal of chemical physics* **2005**, *122* (4), 044507.
(12) Russell, E. R. *Structure and properties of charged colloidal systems*; Harvard University, 2014.
(13) Bergström, L. Hamaker constants of inorganic materials. *Advances in colloid and interface science* **1997**, *70*, 125-169.
(14) Hunter, R. J. *Zeta potential in colloid science: principles and applications*; Academic press, 2013.
(15) Verwey, E. J. W. Theory of the stability of lyophobic colloids. *The Journal of Physical Chemistry* **1947**, *51* (3), 631-636.
(16) Pashley, R. Hydration forces between mica surfaces in aqueous electrolyte solutions. *Journal of colloid and interface science* **1981**, *80* (1), 153-162.
(17) Prakash, A.; Pfaendtner, J.; Chun, J.; Mundy, C. J. Quantifying the molecular-scale aqueous response to the mica surface. *The Journal of Physical Chemistry C* **2017**, *121* (34), 18496-18504.
(18) Zhuravlev, L. The surface chemistry of amorphous silica. Zhuravlev model. *Colloids and Surfaces A: Physicochemical and Engineering Aspects* **2000**, *173* (1-3), 1-38.
(19) Sindorf, D. W.; Maciel, G. E. Cross-polarization magic-angle-spinning silicon-29 nuclear magnetic resonance study of silica gel using trimethylsilane bonding as a probe of surface geometry and reactivity. *The Journal of Physical Chemistry* **1982**, *86* (26), 5208-5219.
(20) Milonjić, S. K. A relation between the amounts of sorbed alkali cations and the stability of colloidal silica. *Colloids and surfaces* **1992**, *63* (1-2), 113-119.

(21) Rimola, A.; Costa, D.; Sodupe, M.; Lambert, J.-F.; Ugliengo, P. Silica surface features and their role in the adsorption of biomolecules: computational modeling and experiments. *Chemical reviews* **2013**, *113* (6), 4216-4313.
(22) Eisenthal, K. Liquid interfaces probed by second-harmonic and sum-frequency spectroscopy. *Chemical reviews* **1996**, *96* (4), 1343-1360.
(23) Onizhuk, M.; Panteleimonov, A.; Kholin, Y. V.; Ivanov, V. Dissociation constants of silanol groups of silic acids: quantum chemical estimations. *Journal of Structural Chemistry* **2018**, *59* (2), 261-271.
(24) Barisik, M.; Atalay, S.; Beskok, A.; Qian, S. Size dependent surface charge properties of silica nanoparticles. *The Journal of Physical Chemistry C* **2014**, *118* (4), 1836-1842.
(25) Alongi, K. S.; Shields, G. C. Theoretical calculations of acid dissociation constants: a review article. *Annual reports in computational chemistry* **2010**, *6*, 113-138.
